# Moyamoya-related intracerebral hemorrhage associated with hyperemesis gravidarum

**DOI:** 10.1177/1753495X251345041

**Published:** 2025-06-03

**Authors:** Elliott B Cohen, Michal Z Sheinis, Frank L Silver, John W Snelgrove

**Affiliations:** 1 Division of Neurology, Department of Medicine, 7938 University of Toronto, Toronto, Canada; 2 Department of Obstetrics and Gynaecology, 7938 University of Toronto, Toronto, Canada; 3 Division of Neurology, University Health Network, 7938 University of Toronto, Toronto, Canada; 4 Department of Obstetrics and Gynaecology, Mount Sinai Hospital, Toronto, Canada

**Keywords:** Moyamoya, intracerebral hemorrhage, stroke, hyperemesis gravidarum

## Abstract

A 32-year-old woman in her first pregnancy, presented with acute-onset hemidystonia and hemianesthesia which occurred while vomiting, on a background of hyperemesis gravidarum. Magnetic resonance imaging of the brain demonstrated right basal ganglia, thalamic, and capsular intracerebral hemorrhage, and time-of-flight magnetic resonance angiography demonstrated an occluded right middle cerebral artery with associated lenticulostriate collateral vessels, consistent with Moyamoya phenomenon. This case highlights the importance of managing hyperemesis gravidarum in patients with Moyamoya phenomenon to avoid Valsalva forces which are associated with intracerebral hemorrhage.

## Case

A healthy 32-year-old right-handed Venezuelan woman, who was experiencing hyperemesis gravidarum, presented to obstetric triage at our hospital several hours after an acute neurological event that occurred while vomiting. She was 16 weeks into her first pregnancy. She reported acute-onset cramping-type pain in her left calf associated with involuntary plantar flexion of her left ankle. Seconds later, she developed acute-onset involuntary flexion of her left elbow. She simultaneously developed acute-onset numbness of the left arm and leg. These symptoms lasted for 5 min but she had persistent mild left arm and leg weakness and intermittent left arm and leg paresthesias. The review of systems was negative.

Neurological examination revealed slowness of repetitive movements of the left arm and hand with finger tapping, fist open-close, and alternating forearm pronation/supination. The left first dorsal interosseous power was graded 4/5 (using the MRC grading scale). There was mild ataxia with finger-to-nose on the left and there was dysdiadochokinesia with rhythmic tapping of the left hand. Gait examination was hesitant due to a subjective sense of left leg weakness. The remainder of her examination was normal.

**Figure 1.**
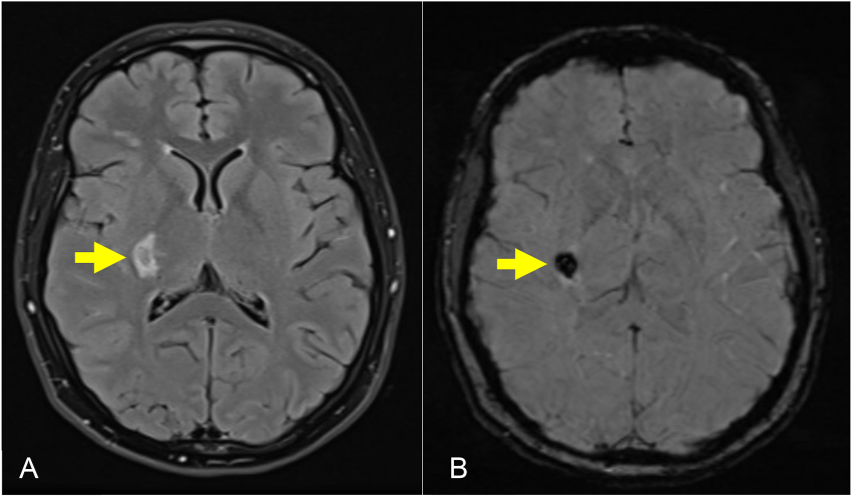
Magnetic resonance imaging (MRI) brain with (a) axial T2 fluid-attenuated inversion recovery (FLAIR) and (b) axial susceptibility-weighted imaging (SWI) sequences demonstrating an intracerebral hemorrhage (ICH) in the right posterior lentiform nucleus, posterior limb of the internal capsule, and lateral thalamus.

**Figure 2.**
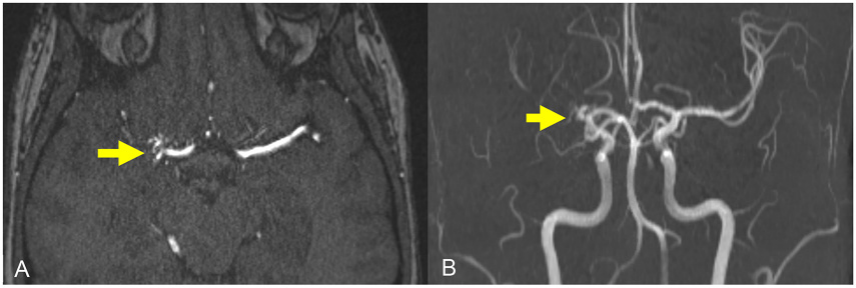
Time-of-flight MR angiogram (TOF MRA) with (a) axial MRA and (b) coronal maximum intensity projection (MIP) sequences demonstrating an occluded right middle cerebral artery (MCA) with small lenticulostriate collateral vessels. [Note that the classic *moyamoya* or “puff of smoke” appearance was described using conventional cerebral angiography; the MRA TOF technique does not readily visualize the smallest collateral vessels and therefore does not produce the same visual effect].

Magnetic resonance imaging of the brain revealed a 2 cm intracerebral hemorrhage (ICH) involving the right posterior lentiform nucleus, lateral thalamus, posterior limb of the internal capsule, and corona radiata (Figure 1). Time-of-flight magnetic resonance angiogram of the circle of Willis revealed a proximally occluded right middle cerebral artery (MCA) with adjacent tortuous lenticulostriate collateral vessels, consistent with a Moyamoya phenomenon (Figure 2). Serum inflammatory markers were negative.

The patient was prescribed an antiemetic to avoid emesis-related Valsalva forces which may elevate the risk of ICH. She was also kept normotensive to prevent the expansion of the hematoma. Her pregnancy was complicated by intrahepatic cholestasis of pregnancy managed with ursodeoxycholic acid but was otherwise unremarkable. She delivered a healthy 4.15 kg baby girl via an uncomplicated cesarean section at 37 + 1 weeks’ gestation. Her postpartum course was uncomplicated and her neurological deficits gradually resolved.

## Discussion

Moyamoya disease is an idiopathic cerebral vasculopathy characterized by progressive stenosis of a proximal intracranial artery with subsequent formation of abnormal collateral vessels. First described in Japan, *moyamoya*, or “puff of smoke” in Japanese, refers to the appearance of the collateral vessels on cerebral angiography.^1^ Pathology of the stenotic vessel wall reveals intimal hyperplasia and fibrosis, while the collateral vessels have a weakened tunica media and contain microaneurysms. The underlying etiology, however, remains unknown. A Moyamoya “phenomenon” can arise secondary to an identifiable etiology such as atherosclerosis, radiation, vasculitis, sickle cell anemia, and neurofibromatosis type 1. Moyamoya disease predominantly affects women in the first four decades of life, coinciding with peak child-bearing years. Patients with Moyamoya disease may present with transient ischemic attack, ischemic or hemorrhagic stroke, seizure, or headache; it may also be discovered incidentally on neuroimaging. There is no cure for Moyamoya disease and treatment is supportive. In Moyamoya-related hemorrhage, reduction of blood pressure is the mainstay of management; however, there is emerging evidence that revascularization surgery may be an option.^2^

The risk of Moyamoya disease–related ICH in pregnant women is similar to that of nonpregnant women with Moyamoya disease.^3^ Therefore, Moyamoya disease is not a contraindication to pregnancy. There is no robust evidence guiding the optimal approach to delivery in these women. Maintaining hemodynamic stability, reducing Valsalva forces, and minimizing pain are critical. The literature to date suggests that these management goals can be achieved via assisted vaginal delivery with epidural anesthesia, and that these women need not necessarily undergo cesarean section.^4,5^ However, these case series included women with a prepregnancy diagnosis of Moyamoya disease without ICH during pregnancy. Therefore, whether the approach to delivery should change in the presence of prepartum ICH remains an unanswered question.

Revisiting our case, the patient's neurological presentation was most in keeping with hemidystonia and hemianesthesia secondary to involvement of the putamen and thalamus, respectively. The persistent weakness and ataxia can be explained by the internal capsule and coronate radiata involvement. The MCA occlusion was chronic as evidenced by the absence of cerebral infarction and the presence of vascular collateralization. A secondary cause for her Moyamoya phenomenon was not discovered.

The Valsalva manoeuvre, which occurs during emesis, transiently increases arterial blood pressure by transmitting raised intrathoracic pressure to the arterial tree and by increasing sympathetic vascular tone.^6^ Indeed, the Valsalva manoeuvre has been reported to be a trigger for ICH.^7^ It is therefore mechanistically plausible that our patient's emesis led to the rupture of a fragile collateral vessel.

To the best of our knowledge, this is the first case report of Moyamoya-related ICH occurring in the context of hyperemesis gravidarum. Healthcare providers caring for pregnant women with a prepregnancy diagnosis of Moyamoya phenomenon should be proactive in treating hyperemesis gravidarum to mitigate the risk of ICH.
